# Clinical and genetic analysis of benign familial infantile epilepsy caused by *PRRT2* gene variant

**DOI:** 10.3389/fneur.2023.1135044

**Published:** 2023-05-09

**Authors:** Yu Gu, Daoqi Mei, Xiaona Wang, Ang Ma, Jinghui Kong, Yaodong Zhang

**Affiliations:** ^1^Department of Pediatrics, Children's Hospital Affiliated to Zhengzhou University, Henan Children's Hospital, Zhengzhou Children's Hospital, Zhengzhou, China; ^2^Department of Neurology, Children's Hospital Affiliated to Zhengzhou University, Henan Children's Hospital, Zhengzhou Children's Hospital, Zhengzhou, China; ^3^Zhengzhou Key Laboratory of Pediatric Neurobehavioral, Henan Neural Development Engineering Research Center, Children's Hospital Affiliated to Zhengzhou University, Zhengzhou, China

**Keywords:** benign familial infantile epilepsy (BFIE), *PRRT2* gene, epilepsy syndrome, heterozygous mutations, anti-seizure medication

## Abstract

**Objective:**

This study presents the clinical phenotypes and genetic analysis of seven patients with benign familial infantile epilepsy (BFIE) diagnosed by whole-exome sequencing.

**Methods:**

The clinical data of seven children with BFIE diagnosed at the Department of Neurology, Children’s Hospital Affiliated to Zhengzhou University between December 2017 and April 2022 were retrospectively analyzed. Whole-exome sequencing was used to identify the genetic causes, and the variants were verified by Sanger sequencing in other family members.

**Results:**

The seven patients with BFIE included two males and five females ranging in age between 3 and 7 months old. The main clinical phenotype of the seven affected children was the presence of focal or generalized tonic–clonic seizures, which was well controlled by anti-seizure medication. Cases 1 and 5 exhibited predominantly generalized tonic–clonic seizures accompanied by focal seizures while cases 2, 3, and 7 displayed generalized tonic–clonic seizures, and cases 4 and 6 had focal seizures. The grandmother and father of cases 2, 6, and 7 had histories of seizures. However, there was no family history of seizures in the remaining cases. Case 1 carried a *de novo* frameshift variant c.397delG (p.E133Nfs*43) in the proline-rich transmembrane protein 2 (*PRRT2*) gene while case 2 had a nonsense variant c.46G > T (p.Glu16*) inherited from the father, and cases 3–7 carried a heterozygous frameshift variant c.649dup (p.R217Pfs*8) in the same gene. In cases 3 and 4, the frameshift variant was *de novo*, while in cases 5–7, the variant was paternally inherited. The c.397delG (p.E133Nfs*43) variant is previously unreported.

**Conclusion:**

This study demonstrated the effectiveness of whole-exome sequencing in the diagnosis of BFIE. Moreover, our findings revealed a novel pathogenic variant c.397delG (p.E133Nfs*43) in the *PRRT2* gene that causes BFIE, expanding the mutation spectrum of *PRRT2*.

## Introduction

Benign familial infantile epilepsy [BFIE; pyridoxine dependent epilepsy, Online Mendelian Inheritance in Man (OMIM) # 605751] is a benign familial neurological disorder with an incidence of 1 in 10,000 (1). Inheritance is autosomal dominant, and the condition is characterized by focal seizures that may progress to secondary generalized tonic–clonic seizures. The age of seizure onset in affected children usually ranges between 4 and 6 months old. The seizures usually occur in clusters and have a good prognosis and usually resolve by 2 years old (2, 3).

Benign familial infantile epilepsy is recognized as a genetically heterogeneous disorder. The *PRRT2* gene, encoding proline-rich transmembrane protein 2, is a major causative gene for BFIE. *PRRT2* located on the short arm 11.2 of chromosome 16, is mainly expressed in the nervous system, especially in the cerebral cortex, hippocampus, basal ganglia, and cerebellum (4, 5). Mutations in *PRRT2* are associated with multiple childhood-onset neurological disorders, including BFIE [(OMIM) # 605751], paroxysmal kinesigenic dyskinesia [PKD; (OMIM) # 128200], and infantile convulsions and choreoathetosis [ICCA; (OMIM) # 602066]. Other genetic mutations, including mutations in *SCN2A*, *KCNQ2*, *SCN8A*, and *ATP1A2*, have also been found to cause BFIE (6). However, these genes do not account for all cases of BFIE and the causative genes in some patients remain unknown. In this study, we summarized the clinical phenotypes of seven affected children from the Chinese Han population diagnosed with BFIE and analyzed the genetic etiologies underlying the disease in these cases.

## Materials and methods

The present study was a case series study and was approved by the Medical Ethics Committee of the Children’s Hospital Affiliated with Zhengzhou University. Informed consent was obtained from the children’s guardians. We retrospectively analyzed the clinical data of seven children diagnosed with BFIE in the neurology outpatient ward of our hospital from December 2017 to April 2022. The diagnosis of BFIE was confirmed by clinical features and genetic diagnosis in all these patients.

The clinical data of seven children were collected. The laboratory tests included routine blood tests, tests for liver, kidney, and thyroid function, blood ammonia, pyruvate, lactate, inorganic elements, nine vitamins, ceruloplasmin, and genetic metabolic screening of the blood and urine. Scale examinations included the pediatric neuropsychological screening scale (DQ), imaging tests included cranial computed tomography (CT), and magnetic resonance imaging (MRI), and electrophysiological tests included long-range video electroencephalogram monitoring.

After obtaining informed consent from the children’s guardians, 2 mL of peripheral venous blood was collected from each child and the parents into ethylenediaminetetraacetic acid tubes. Whole-exome sequencing was performed on the three members of each family and the suspected variants with clinical significance were verified in the family members using Sanger sequencing. Genetic sequencing was performed by the Beijing Zhiyin Oriental Translational Medicine Research Center Co., Ltd., and the relevant data analysis was conducted by Henan Provincial Key Laboratory of Children’s Genetics and Metabolic Diseases in our hospital.

## Results

### Clinical characteristics

**Figure 1:**
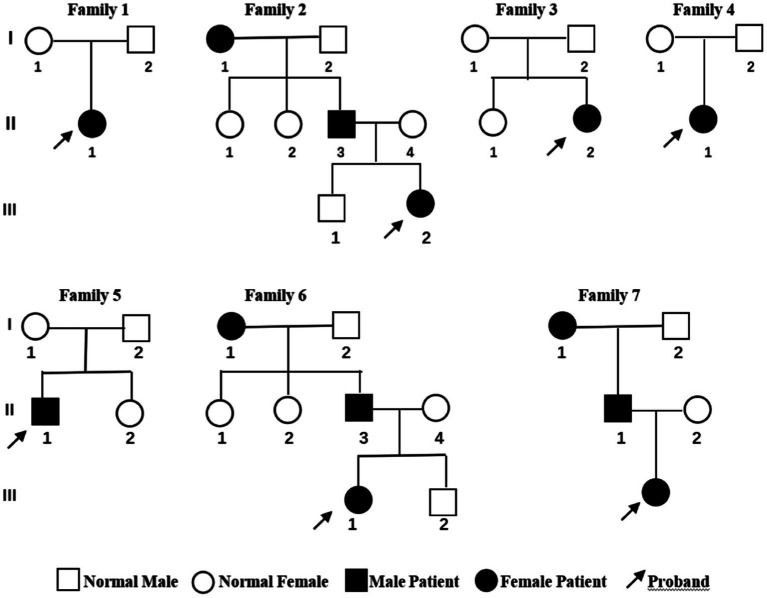
Genetic pedigree of the seven children with benign familial infantile epilepsy. Squares represent males. Circles indicate females. Black indicates a history of epilepsy. The arrows indicate the proband in the family.

All the children experienced seizures of varying severity during infancy; details of the clinical manifestations of the children are listed in Table 1. Cases 1 and 5 mainly displayed generalized tonic–clonic seizures accompanied by focal seizures, cases 2, 3, and 7 mainly experienced generalized tonic–clonic seizures, and cases 4 and 6 showed mainly focal seizures. All the cases were effectively controlled by anti-epileptic drug treatment. The parents of the seven children were non-consanguineous. None of the parents of cases 1, 3, 4, or 5 had a history of seizures, whereas the grandmother and father of cases 2, 6, and 7 had a history of seizures. In addition, cases 3 and 5 each had a sister, and cases 2 and 6 brothers, and none of these siblings had a history of seizures (Figure 1).

**Table 1:** Clinical data of seven children with benign familial infantile convulsions.

ID/Sex	Age^1^	Birth history	Age^2^ of onset	Types of seizures	Initial physical examination	Video EEG^3^	Head MRI^4^	Efficacy and follow-up	Evolution
1F^5^	6 months 15 days	G1P1, full term cesarean section, W: 2.8 kg, no history of asphyxia or resuscitation	4 months	(1) GTCS^6^; (2) focal seizures	W: 7 kg, HC^7^: 42 cm, fontanelle 1.5 × 2.0 cm; normal pursuit of vision and hearing; vertical head stability; and unable to sit alone	Abnormal	Delayed myelination, and bilateral frontotemporal subarachnoid space widened	LEV^8^	Control
2M^9^	4 months 13 days	G2P2 full term cesarean section, W: 3.0 kg, no history of hypoxia or asphyxia	3 months and 13 days	GTCS	W: 6 kg, HC: 39 cm, vertical head stability; poor pursuit response	Abnormal	Bilateral frontotemporal subarachnoid space widened	LEV, vitamin B6	Improvement (seizures reduced)
3F	4 months 20 days	G2P2, 38^+5^ weeks, cesarean section, W: 3.9 kg, no history of asphyxia or resuscitation	4 months and 9 days	Generalized seizure on awakening	W: 7 kg, HC: 40.5 cm, fontanelle 0.5 × 0.5 cm, head raised steadily; normal muscle strength and tone	Normal	Normal	VPA^10^ → PB^11^	Control
4F	6 months 5 days	G1P1, 39 weeks normal delivery, W: 3.2 kg, no history of asphyxia or choking	6 months	Focal seizures	W: 8 kg, fontanelle 1.5 × 1.5 cm, head raised steadily; unstable sit; normal pursuit of vision and hearing; normal muscle strength and tone	Abnormal	Delayed myelination	OXC^12^	Control
5 M	5 months	G2P2, delivered at 39^+3^ weeks, W: 3.85 kg, no history of perinatal hypoxic asphyxia	4 months 10 days	(1) GTCS; (2) Focal seizures	W: 7 kg, HC: 41 cm, fontanelle 1.5 × 1.5 cm, poor tracking vision and hearing; head raised steadily at 3 months, turn over at 4 months	Abnormal	Normal	LEV, vitamin B6 tablets → OXC	Control
6F	4 months 9 days	G2P2, full term cesarean section, W: 3.85 kg, no history of perinatal hypoxic asphyxia	4 months and 3 days	Focal seizures	W: 7.5 kg, HC: 40 cm, fontanelle 1.0 × 1.0 cm	Normal	Bilateral frontotemporal subarachnoid space widened	LEV, vitamin B6 tablets → OXC	Control
7F	5 months 15 days	G2P2, full term normal birth, W: 3.6 kg, no history of asphyxia or resuscitation	5 months 10 days	GTCS	W: 7 kg, HC: 43 cm, fontanelle 2.0 × 1.5 cm, head raised steadily, normal hearing and smiling, normal muscle strength and tone	Normal	Normal	LEV, PB	Control

^1^Age, current age; ^2^Age of onset, Age of first onset; ^3^Video EEG, Video EEG features; ^4^Head MRI, Head magnetic Resonance Imaging; ^5^Female; ^6^GTCS, generalized tonic–clonic seizure; ^7^HC, head circumference; ^8^LEV, levetiracetam; ^9^M, Male; ^10^VPA, valproic acid; ^11^PB, phcnobarbital; and ^12^OXC, oxcarbazepine.

**Figure 2:**
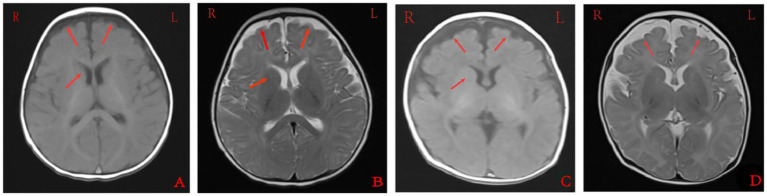
Cranial magnetic resonance imaging findings of case 1 **(A,B)** and case 2 **(C,D)**. **A** and **C** were T1 sequences, showed low signal in the widening of bilateral frontotemporal subarachnoid space, high signal in T1WI of the bilateral inner capsule forelimb, and delayed myelin sheath development compared to children of the same age. **B** and **D** were T2 sequences that showed high signal in the widening of the bilateral frontotemporal subarachnoid space, and low and fuzzy signal on T2WI of the bilateral inner capsule forelimbs. EEG: 155 new1; Amplitude: 100 μV/cm; Low frequency: 0.3 s; High frequency: 15 Hz; Trapped wave: 50 Hz; and Multi speed: 3.0 cm/s.

**Figure 3:**
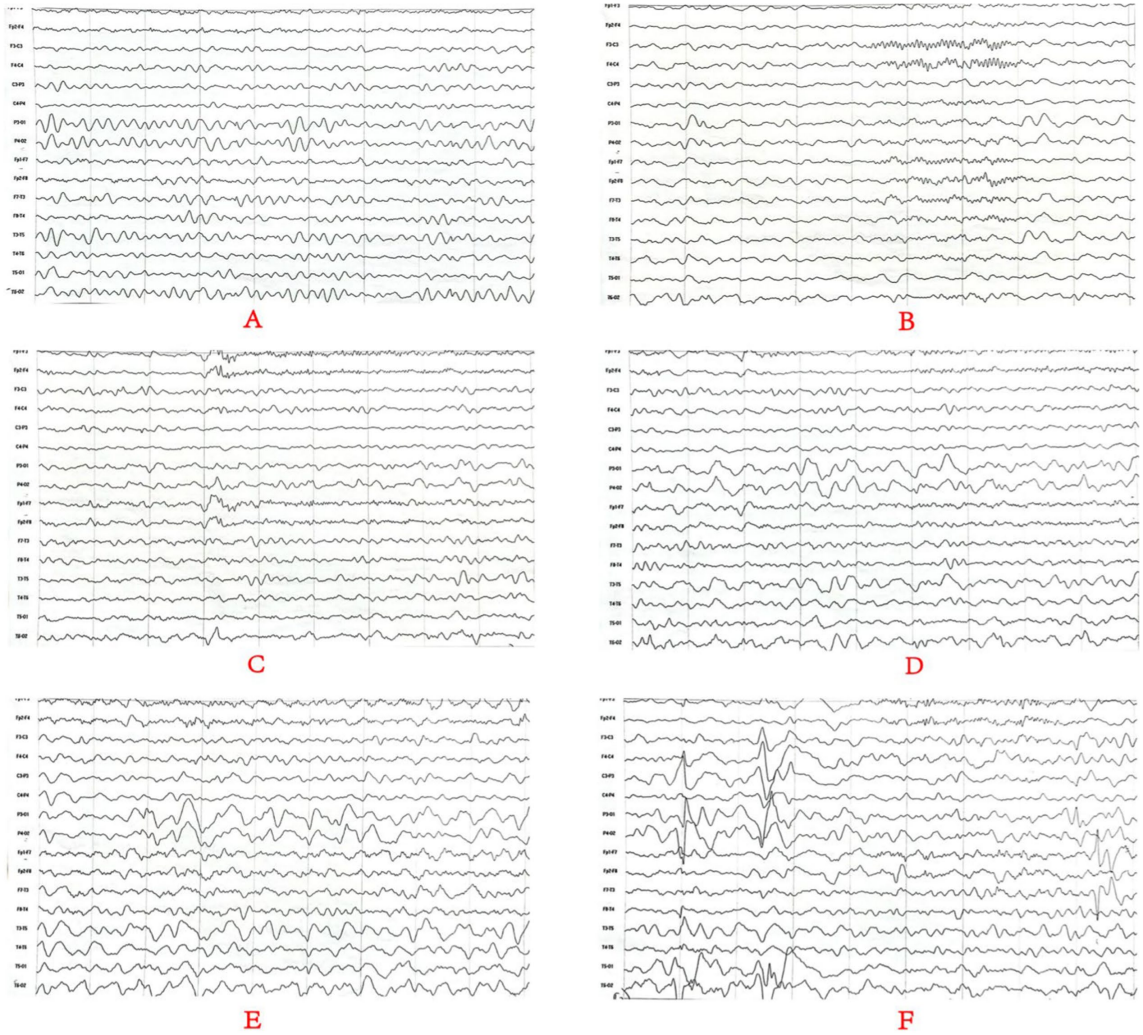
Monitoring results for the long-range video electroencephalogram of case 4. Panel **(A)** is background EEG with low-medium amplitude θ activity in the bilateral occipital area at 5–6 Hz; Panel **(B)** is the EEG in the sleep stage; Panels **(C–E)** is the EEG in the attack stage and **(F)** is the EEG at the end of the attack stage. Two focal onset attacks were recorded in waking stage with a simultaneous abnormal low-medium sharp wave and sharp slow waves well as a simultaneous amplitude sharp wave and sharp slow wave.

Cranial MRI showed varying degrees of cerebral white matter hemi-oval central myelin hypoplasia at 6 months old in cases 1 and 4, indicating a delay in neuronal development. On the other hand, the cranial MRI showed varying degrees of frontotemporal subarachnoid widening in cases 1, 2, and 6 (Figure 2). No developmental delays in terms of growth and cognitive function compared with normal children of the same age were observed in any of the seven cases. Video electroencephalograph (EEG) testing in cases 1, 2, 4, and 5 showed varying degrees of abnormal discharge (Figure 3).

### Genetic analysis

**Figure 4:**
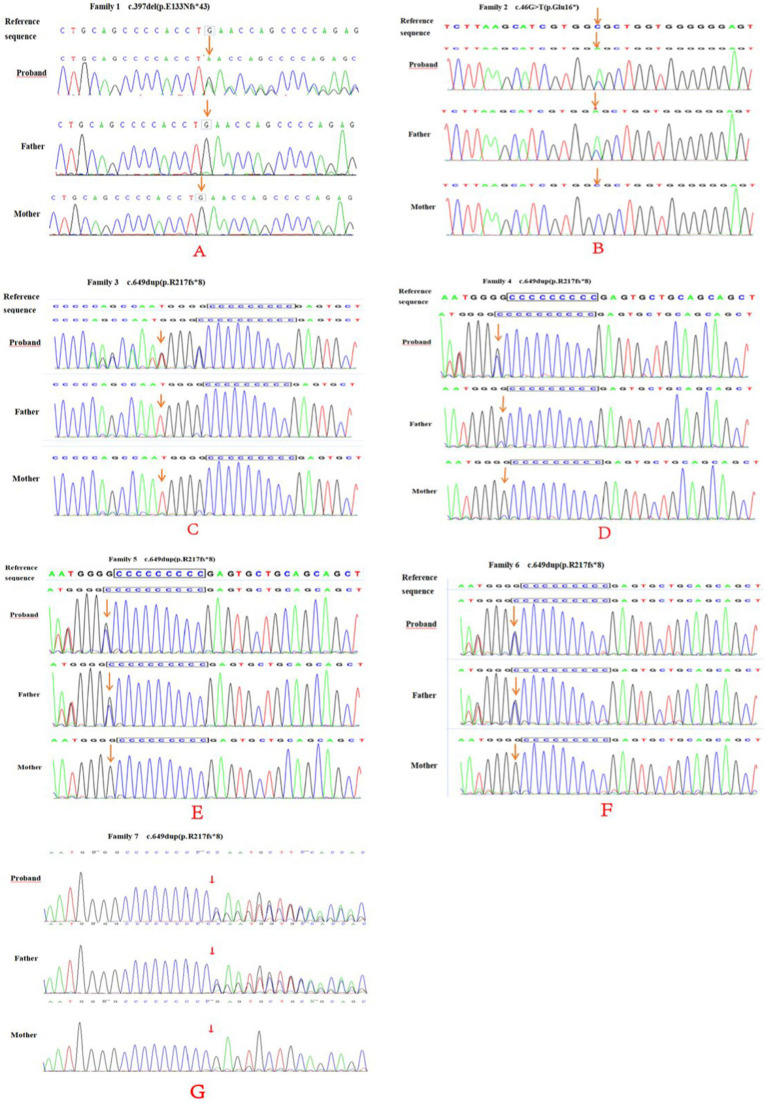
PRRT2 gene sequencing of family 1–7 **(A–E)** and their parents. Family 1 **(A)** was c.397del (p.E133Nfs*43), a frame-shift newborn mutation (arrow). No mutation was found in either parents (arrow). Family 2 **(B)** had a c.46G > T (p.Glu16*) nonsense mutation (arrow) and the father had a heterozygous mutation (arrow). Family 3 **(C)** and 4 **(D)** and 4 were c.649dup (p.R217fs*8) newborn frame-shift variation (arrow), and no mutations were found in either parents (arrow). Family 5–7 **(E–G)** and their parents showed a c.649dup (p.R217fs*8) frame-shift variation (arrow), and heterozygous variation locus for the father (arrow).

Table 2 summarizes variants observed in the *PRRT2* (NM_145239.2) gene in the seven children. Whole-exome sequencing analysis showed that among the seven probands, cases 2, 5, 6, and 7 carried variants inherited from the father, while the variants in cases 1, 3, and 4 were *de novo*. Case 1 carried a *de novo* frameshift shift variant c.397delG (p.E133Nfs*43; Figure 4), case 2 carried a nonsense variant c.46G > T (p.Glu16*), cases 3 and 4 had *de novo* frameshift variants c.649dup (p.R217Pfs*8), and all the variants in cases 5, 6, and 7 were frameshift variants c.649dup (p.R217Pfs*8). The c.397delG (p.E133Nfs*43) frameshift variant was novel and has not been reported previously. The evidence for the pathogenicity of c.397delG (p.E133Nfs*43) included PVS1, PS2, PM2, and PP3. The variant was predicted to be deleterious by multiple software programs including SIFT, Polyphen-2, and MutationTaster. According to the standards and guidelines of the ACMG (7), c.397delG (p.E133Nfs*43) was classified as a pathogenic variant. The c.649dup (p.R217Pfs*8) and c.46G > T(p. Glu16*) variants are known pathogenic variants, as reported in previous studies (8–10).

**Table 2:** Analysis of the PRRT2 gene variants in seven cases as follows.

Sequence	1	2	3	4	5	6	7
PRRT2 mutation	c.397delG (p.E133Nfs*43)	c.46G > T (p.Glu16*)	c.649dup(p.R217Pfs*8)	c.649dup(p.R217Pfs*8)	c.649dup(p.R217Pfs*8)	c.649dup(p.R217Pfs*8)	c.649dup(p.R217Pfs*8)
ACMG Rating	PVS1 + PS2 + PM2	PVS1 + PS2 + PM2	PVS1 + PS2 + PS4 + PP1_Strong	PVS1 + PS2 + PS4 + PP1_Strong	PVS1 + PS4 + PP1_Strong	PVS1 + PS4 + PP1_Strong	PVS1 + PS4 + PP1_Strong
Pathogenicity analysis	Pathogenic	Pathogenic	Pathogenic	Pathogenic	Pathogenic	Pathogenic	Pathogenic
Type of variation	Shift code *de novo* variant, wild type parents	Nonsense variant, source father heterozygous	Shift code *de novo* variant, wild type parents	Shift code *de novo* variant, wild type parents	Shift code variation, source father heterozygosity	Shift code variation, source father heterozygosity	Shift code variation, source father heterozygosity
Source, Phenotype	Parents without phenotype	Father has phenotype	Parents without phenotype	Parents without phenotype	Parents without phenotype	Father has phenotype	Father has phenotype

ACMG, American College of Medical Genetics and Genomics; PVS1, very strong evidence of pathogenicity; PS1, strong evidence 1 of pathogenicity; PS2, strong evidence 2 of pathogenicity; PM1, moderate evidence 1 of pathogenicity; PM2, moderate evidence 2 of pathogenicity; PP3, supporting evidence 3 of pathogenicity; and PP5, supporting evidence 5 of pathogenicity.

## Discussion

Benign familial infantile epilepsy is an autosomal dominant epilepsy that was first reported by Vigevano et al. (11) and was named BFIE in 2010 by the International League Against Epilepsy (ILAE) (12). The main clinical criteria for diagnosis (13) include (1) first onset at 3–12 months old, (2) family history of benign infantile epilepsy, (3) normal psychomotor development before and after onset, (4) focal seizures, alone or followed by generalized seizures, with ≥2 seizures within 24 h, mostly cluster seizures, usually without persistent status epilepticus, (5) normal EEG background during interictal periods with Rolandic epilepsy, (6) no abnormalities in cranial imaging, (7) exclusion of convulsions due to metabolic disorders such as hypocalcemia and hypoglycemia, and (8) self-limiting seizures or seizures that respond well to antiepileptic drugs, with resolution before the age of 2 years old (14, 15). In this study, all the seven affected children were within 3–7 months old, and some of them had a family history of seizure disorders. Moreover, cases 1 and 5 mainly displayed generalized tonic—clonic seizures accompanied by focal seizures, cases 2, 3, and 7 mainly exhibited generalized tonic—clonic seizures, and cases 4 and 6 mainly displayed focal seizures. However, several of the cases in this study were found to have varying degrees of myelin dysplasia and widening of the frontotemporal subarachnoid space on cranial MRI testing.

Multiple causative genes associated with BFIE have been reported, including *PRRT2*, *SCN2A*, *KCNQ2*, *SCN8A, ATP1A2, KCNA1*, *KCNMA1*, *BFIE1*, and *BFIE4* (2, 6). *PRRT2* encodes an ion channel and was found to be a major causative gene for BFIE by Heron et al. (16). The *PRRT2* gene, located on chromosome 16p11.2, consists of four exons and encodes a protein containing 340 amino acids (17, 18). The PRRT2 protein consists of a proline-rich N-terminal sequence (N-glycosylation site), two transmembrane structural domains, and a C-terminal sequence. The transmembrane region is highly conserved and has important physiological functions (4, 19–21). *PRRT2* is mainly expressed in the presynaptic membrane and cytoplasm of neurons in the cerebral cortex, basal ganglia, cerebellum, and hippocampus. The PRRT2 protein plays a key role in neurotransmitter release by interacting with fusion complexes and calcium sensor proteins involved in synaptic vesicle cytokinesis and calcium sensitivity. Functional analysis showed that *PRRT2* knockout in excitatory neurons resulted in slowed cytokinesis kinetics, reduced synaptic transmission, and significantly increased susceptibility to chemotaxis. In neuronal networks, deletion of *PRRT2* was found to lead to increased spontaneous and evoked activity, resulting in dysregulation of neuronal excitability in various regions of the brain, ultimately triggering paroxysmal movement disorders and seizures (8). All of the seven patients in the present study showed seizures of varying degrees. Cases 1 and 5 had predominantly generalized tonic–clonic seizures together with focal seizures, cases 2, 3, and 7 had generalized tonic–clonic seizures, while cases 4 and 6 had focal seizures. While the grandmother and father of cases 2, 6, and 7 had a history of seizures, there was no family history of seizures in the remaining cases (Figure 1).

According to the Human Genome Variation Society (HGVS), nearly 100 variants have been reported in the *PRRT2* gene, including missense, nonsense, frameshift, splice site, deletion, and insertion variants, with the highest proportion of frameshift variants occurring mainly in exon 2, resulting in truncation and decay of the expressed protein (2). Among the *PRRT2* variants, c.649dupC is by far the most common cause of BFIE, accounting for nearly 80% of cases (8, 9, 22). In this study, all seven affected children carried heterozygous variants in exon 2 with one of the known pathogenic variants, c.649dup (p.R217Pfs*8), accounting for 71.4% (5/7) of the cases, consistent with previous reports (8, 9). Case 1 carried an unreported variant, c.397delG (p.E133Nfs*43), which was predicted to be deleterious and pathogenic by multiple software programs. Luo et al. (3) reported that seven family members carrying heterozygous mutations in the *PRRT2* gene had no clinical symptoms associated with *PRRT2*-related disorders, suggesting incomplete penetrance of the *PRRT2* mutations. In the current study, the variant in case 5 was inherited from the father who showed no clinical phenotype, also suggesting incomplete penetrance.

Proline-rich transmembrane protein 2 has analogs in various vertebrate species, such as humans, gorillas, macaques, and mice, whereas no homologs have been found in invertebrates such as nematodes (2, 4). In humans and rodents, PRRT2 is a neuroprotein that is most abundantly expressed in the cerebellum, basal ganglia, and neocortex. Mutations in *PRRT2* are associated with a variety of neurological disorders, such as BFIE, paroxysmal kinesigenic dyskinesia, and infantile convulsions and choreoathetosis, which account for more than 90% of all cases (3, 23). Other rare phenotypes, including seizures, ictal ataxia, and hemiplegic migraine, have also been reported, suggesting significant phenotypic heterogeneity resulting from *PRRT2* mutations (24–26). To date, most *PRRT2* mutations have been labeled “benign” and lead to self-limited familial infantile epilepsy. However, a small number of patients with *PRRT2* variants have been reported to exhibit severe neurological deficits, such as focal seizures and epileptic spasms, severe seizures, cognitive impairment, or complex malformations (27, 28). In general, the genotype—phenotype correlation of *PRRT2* mutations remains unclear, and there are numerous genetic variants and loci with no direct correlation between genotype and clinical phenotype. In addition to BFIE, mutations in *PRRT2* also cause paroxysmal kinesigenic dyskinesia (PKD), with a prevalence estimated at 1:150,000, characterized by recurrent episodes, transient chorea, dystonia, and/or ballismus (18). In the present study, none of the seven affected children or their family members showed any signs of PKD. Nevertheless, the development of PKD at a later stage cannot be ruled out, as the children are young. Long-term follow-up might be required to monitor the possible development of PKD.

Cranial MRI is usually nonspecific for BFIE as some patients appear normal while others show diffuse hypomyelination, a thin corpus callosum, or high signals in the basal ganglia, thalamus, or hippocampus (29). In this study, cranial MRI showed no abnormal brain changes in cases 3, 5, and 7 while in cases 1 and 4, the development of white-matter myelination was delayed. Moreover, cases 2 and 6 displayed varying degrees of widening of the subarachnoid space (Figure 2). Furthermore, previous studies have shown that interictal EEGs in BFIE are usually normal, though some BFIEs may exhibit interictal focal epileptiform discharges, mostly originating in the parieto-occipital lobe and located in the frontotemporal region (12, 30, 31). Here, we found that cases 3, 6, and 7 had no abnormal discharges on long-range video in EEG monitoring, whereas cases 1, 2, 4, and 5 displayed focal discharges of varying degrees during the interictal period. Cases 1, 2, and 5 had discharges in the frontotemporal region, consistent with previous studies (31).

In terms of treatment, most children with BFIE respond well to antiepileptic drugs, and seizures are usually completely controlled by 2 years old (9). Several studies (32) have shown that in some BFIE patients, initial treatment regimens of levetiracetam were not effective, and seizures were controlled by switching to oxcarbazepine or sodium valproate. Additionally, oxcarbazepine has fewer adverse effects and no effect on cognitive function. In the present study, the seven affected children underwent treatment and follow-up. Case 1 was well-controlled with levetiracetam while case 4 was treated with oxcarbazepine alone and remained seizure-free. Seizure control was achieved in case 3 using sodium valproate combined with phenobarbital. Although treatment with levetiracetam resulted in poor control in the remaining four cases, complete control was achieved after switching to oxcarbazepine, which is consistent with the findings of previous studies (32).

Early epilepsy (whether secondary or systemic) is representative of a number of disorders, often with devastating and persistent adverse consequences. Many brain malformations and inborn metabolic disorders are caused by genetic factors, such as ion channel disease, which may be associated with abnormalities in brain structure. Most children with neurometabolic disorders show some signs of disordered metabolism, which can be differentially diagnosed by genetic testing. When the diagnostic criteria are unclear, genetic testing may be the most effective means of diagnosing these diseases. Moreover, genetic testing can also guide the application of appropriate antiepileptic drugs and clinical management (33, 34). In the current study, the seizures were controlled within 2 years of age and there has been no recurrence so far in the seven affected children. In addition, the growth and language development of the seven children have been normal, and their muscle tone is normal. These results indicate that genetic testing is beneficial in the clinical diagnosis and treatment of BFIE.

## Conclusion

In summary, BFIE is a genetic epilepsy with onset in the first year of life. *PRRT2* is a major causative gene of BFIE, with mutations in the gene showing an expanding clinical spectrum and incomplete penetrance. Genetic testing is critical for the diagnosis and clinical management of BFIE patients and is beneficial for prognostic prediction. Moreover, the current study identified a novel BFIE-associated variant, c.397delG (p.E133Nfs*43), in the *PRRT2* gene, thereby expanding the genetic spectrum of BFIE.

## Data availability statement

The datasets presented in this study can be found in online repositories. The names of the repository/repositories and accession number(s) can be found at: https://www.ncbi.nlm.nih.gov/, SCV002760034.

## Ethics statement

Written informed consent was obtained from the minor(s)’ legal guardian/next of kin for the publication of any potentially identifiable images or data included in this article.

## Author contributions

YG, DM, YZ, XW, AM, and JK contributed to the study conception and design and performed material preparation and data collection and analysis. The first draft of the manuscript was written by YG, DM, and YZ. XW and YZ critically revised the manuscript. All authors contributed to the article and approved the submitted version.

## Funding

This work was supported by Joint Construction Project of Henan Medical Science and Technology Project (LHGJ20200618, 2018020633, and LHGJ20200640), Scientific and Technological Project of Henan (212102310034, 232102311006, and 232102310077), Henan Engineering Research Center of Childhood Neurodevelopment Open Project (SG201907), and National Natural Science Foundation of China (81901387).

## Conflict of interest

The authors declare that the research was conducted in the absence of any commercial or financial relationships that could be construed as a potential conflict of interest.

## Publisher’s note

All claims expressed in this article are solely those of the authors and do not necessarily represent those of their affiliated organizations, or those of the publisher, the editors and the reviewers. Any product that may be evaluated in this article, or claim that may be made by its manufacturer, is not guaranteed or endorsed by the publisher.

## References

[1] Symonds JD Zuberi SM Stewart K McLellan A O‘Regan M MacLeod S. Incidence and phenotypes of childhood-onset genetic epilepsies: a prospective population-based national cohort. Brain. (2019) 142:2303–18. doi: 10.1093/brain/awz195, PMID: 31302675 PMC6658850

[2] He J Tang H Liu C Tan L Xiao W Xiao B. Novel PRRT2 gene variants identified in paroxysmal kinesigenic dyskinesia and benign familial infantile epilepsy in Chinese families. Exp Ther Med. (2021) 21:504. doi: 10.3892/etm.2021.9935, PMID: 33791013 PMC8005681

[3] Luo HY Xie LL Hong SQ Li XJ Li M Hu Y. The genotype and phenotype of proline-rich transmembrane protein 2 associated disorders in chinese children. Front Pediatr. (2021) 9:676616. doi: 10.3389/fped.2021.676616, PMID: 34041212 PMC8141857

[4] Yang L You C Qiu S Yang X Li Y Liu F. Novel and de novo point and large microdeletion mutation in PRRT2-related epilepsy. Brain Behav. (2020) 10:e01597. doi: 10.1002/brb3.1597, PMID: 32237035 PMC7218244

[5] Vlaskamp DRM Callenbach PMC Rump P Giannini LAA Brilstra EH Dijkhuizen T. PRRT2-related phenotypes in patients with a 16p11.2 deletion. Eur J Med Genet. (2019) 62:265–9. doi: 10.1016/j.ejmg.2018.08.002, PMID: 30125676

[6] Zara F Specchio N Striano P Robbiano A Gennaro E Paravidino R. Genetic testing in benign familial epilepsies of the first year of life: clinical and diagnostic significance. Epilepsia. (2013) 54:425–36. doi: 10.1111/epi.12089, PMID: 23360469

[7] Richards S Aziz N Bale S Bick D das S Gastier-Foster J. Standards and guidelines for the interpretation of sequence variants: a joint consensus recommendation of the American college of medical genetics and genomics and the association for molecular pathology. Genet Med. (2015) 17:405–24. doi: 10.1038/gim.2015.30, PMID: 25741868 PMC4544753

[8] Döring JH Saffari A Bast T Brockmann K Ehrhardt L Fazeli W. The phenotypic spectrum of PRRT2-associated paroxysmal neurologic disorders in childhood. Biomedicine. (2020) 8:456. doi: 10.3390/biomedicines8110456, PMID: 33126500 PMC7719266

[9] Zhao Q Liu Z Hu Y Fang S Zheng F Li X. Different experiences of two PRRT2-associated self-limited familial infantile epilepsy. Acta Neurol Belg. (2020) 120:1025–8. doi: 10.1007/s13760-020-01348-9, PMID: 32246320 PMC7383030

[10] Kita M Kuwata Y Murase N Akiyama Y Usui T. A novel truncation mutation of the PRRT2 gene resulting in a 16-amino-acid protein causes self-inducible paroxysmal kinesigenic dyskinesia. Mov Disord Clin Pract. (2017) 4:625–8. doi: 10.1002/mdc3.12500, PMID: 30713971 PMC6353529

[11] Vigevano F Fusco L Di Capua M Ricci S Sebastianelli R Lucchini P. Benign infantile familial convulsions. Eur J Pediatr. (1992) 151:608–12. doi: 10.1007/bf01957732 1505581

[12] Berg AT Berkovic SF Brodie MJ Buchhalter J Cross JH van Emde BW. Revised terminology and concepts for organization of seizures and epilepsies: report of the ILAE commission on classification and terminology, 2005-2009. Epilepsia. (2010) 51:676–85. doi: 10.1111/j.1528-1167.2010.02522.x, PMID: 20196795

[13] Vigevano F. Benign familial infantile seizures. Brain Dev. (2005) 27:172–7. doi: 10.1016/j.braindev.2003.12.012 15737697

[14] Lu JG Bishop J Cheyette S Zhulin IB Guo S Sobreira N. A novel PRRT2 pathogenic variant in a family with paroxysmal kinesigenic dyskinesia and benign familial infantile seizures. Cold Spring Harb Mol Case Stud. (2018) 4:a002287. doi: 10.1101/mcs.a002287, PMID: 29167286 PMC5793775

[15] Zhao SY Li LX Chen YL Chen YJ Liu GL Dong HL. Functional study and pathogenicity classification of PRRT2 missense variants in PRRT2-related disorders. CNS Neurosci Ther. (2020) 26:39–46. doi: 10.1111/cns.13147, PMID: 31124310 PMC6930815

[16] Heron SE Grinton BE Kivity S Afawi Z Zuberi SM Hughes JN. PRRT2 mutations cause benign familial infantile epilepsy and infantile convulsions with choreoathetosis syndrome. Am J Hum Genet. (2012) 90:152–60. doi: 10.1016/j.ajhg.2011.12.003, PMID: 22243967 PMC3257886

[17] Lee EH. Epilepsy syndromes during the first year of life and the usefulness of an epilepsy gene panel. Kor J Pediatr. (2018) 61:101–7. doi: 10.3345/kjp.2018.61.4.101, PMID: 29713355 PMC5924840

[18] Hatta D Shirotani K Hori Y Kurotaki N Iwata N. Activity-dependent cleavage of dyskinesia-related proline-rich transmembrane protein 2 (PRRT2) by calpain in mouse primary cortical neurons. FASEB J. (2020) 34:180–91. doi: 10.1096/fj.201902148R, PMID: 31914621

[19] Landolfi A Barone P Erro R. The spectrum of PRRT2-associated disorders: update on clinical features and pathophysiology. Front Neurol. (2021) 12:629747. doi: 10.3389/fneur.2021.629747, PMID: 33746883 PMC7969989

[20] El Achkar CM Rosen Sheidley B O'Rourke D Takeoka M Poduri A. Compound heterozygosity with PRRT2: pushing the phenotypic envelope in genetic epilepsies. Epilepsy Behav Case Rep. (2019) 11:125–8. doi: 10.1016/j.ebcr.2016.12.001, PMID: 31193310 PMC6525261

[21] Baldi S Zhu JL Hu QY Wang JL Zhang JB Zhang SH. A novel PRRT2 variant in chinese patients suffering from paroxysmal kinesigenic dyskinesia with infantile convulsion. Behav Neurol. (2020) 2020:2097059. doi: 10.1155/2020/2097059, PMID: 32509037 PMC7251426

[22] Heron SE Dibbens LM. Role of PRRT2 in common paroxysmal neurological disorders: a gene with remarkable pleiotropy. J Med Genet. (2013) 50:133–9. doi: 10.1136/jmedgenet-2012-101406, PMID: 23343561

[23] Zeng Q Yang X Zhang J Liu A Yang Z Liu X. Genetic analysis of benign familial epilepsies in the first year of life in a Chinese cohort. J Hum Genet. (2018) 63:9–18. doi: 10.1038/s10038-017-0359-x, PMID: 29215089 PMC8075886

[24] Fay AJ McMahon T Ima C Bair-Marshall C Niesner KJ Li H. Age-dependent neurological phenotypes in a mouse model of PRRT2-related diseases. Neurogenetics. (2021) 22:171–85. doi: 10.1007/s10048-021-00645-6, PMID: 34101060 PMC8241743

[25] Rochette J Roll P Fu YH Lemoing AG Royer B Roubertie A. Novel familial cases of ICCA (infantile convulsions with paroxysmal choreoathetosis) syndrome. Epileptic Disord. (2010) 12:199–204. doi: 10.1684/epd.2010.0328, PMID: 20716510

[26] Becker F Schubert J Striano P Anttonen AK Liukkonen E Gaily E. PRRT2-related disorders: further PKD and ICCA cases and review of the literature. J Neurol. (2013) 260:1234–44. doi: 10.1007/s00415-012-6777-y, PMID: 23299620

[27] Pavone P Corsello G Cho SY Pappalardo XG Ruggieri M Marino SD. PRRT2 gene variant in a child with dysmorphic features, congenital microcephaly, and severe epileptic seizures: genotype-phenotype correlation? Ital J Pediatr. (2019) 45:159. doi: 10.1186/s13052-019-0755-2, PMID: 31801583 PMC6894132

[28] Ebrahimi-Fakhari D Saffari A Westenberger A Klein C. The evolving spectrum of PRRT2-associated paroxysmal diseases. Brain. (2015) 138:3476–95. doi: 10.1093/brain/awv317, PMID: 26598493

[29] Pisano T Numis AL Heavin SB Weckhuysen S Angriman M Suls A. Early and effective treatment of KCNQ2 encephalopathy. Epilepsia. (2015) 56:685–91. doi: 10.1111/epi.12984 25880994

[30] Zorzi G Conti C Erba A Granata T Angelini L Nardocci N. Paroxysmal dyskinesias in childhood. Pediatr Neurol. (2003) 28:168–72. doi: 10.1016/s0887-8994(02)00512-x 12770667

[31] van Strien TW van Rootselaar AF Hilgevoord AA Linssen WH Groffen AJ Tijssen MA. Paroxysmal kinesigenic dyskinesia: cortical or non-cortical origin. Parkinsonism Relat Disord. (2012) 18:645–8. doi: 10.1016/j.parkreldis.2012.03.006 22464846

[32] Callenbach PM de Coo RF Vein AA Arts WF Oosterwijk J Hageman G. Benign familial infantile convulsions: a clinical study of seven dutch families. Eur J Paediatr Neurol. (2002) 6:269–83. doi: 10.1053/ejpn.2002.0609, PMID: 12374579

[33] Berg AT Coryell J Saneto RP Grinspan ZM Alexander JJ Kekis M. Early-life epilepsies and the emerging role of genetic testing. JAMA Pediatr. (2017) 171:863–71. doi: 10.1001/jamapediatrics.2017.1743, PMID: 28759667 PMC5710404

[34] McKnight D Morales A Hatchell KE Bristow SL Bonkowsky JL Perry MS. Genetic testing to inform epilepsy treatment management from an international study of clinical practice. JAMA Neurol. (2022) 79:1267–76. doi: 10.1001/jamaneurol.2022.3651, PMID: 36315135 PMC9623482

